# 570 Burn-Related Injuries Treated at Two Gulf Coast Hospitals During Following a Category 4 Hurricane

**DOI:** 10.1093/jbcr/irac012.198

**Published:** 2022-03-23

**Authors:** Nicole M Kopari, Mario Rivera, Herb A Phelan, Randy D Kearns, Jeffrey E Carter

**Affiliations:** Children's Hospital New Orleans, New Orleans, Louisiana; University Medical Center New Orleans Burn Center, New Orleans, Louisiana; LSUHSC-New Orleans Department of Surgery, New Orleans, Louisiana; University of New Orleans, New Orleans, Louisiana; University Medical Center- New Orleans, New Orleans, Louisiana

## Abstract

**Introduction:**

Natural disasters are commonly associated with mass destruction and severe injuries. On August 29th, 2021 a category 4 hurricane made landfall before mandatory evacuations were ordered in a major metropolitan community. The powerful storm challenged disaster management teams and first responders as communities struggled to recover. Our study analyzes the demographics of those injured and the injury patterns treated at our state’s only verified burn/trauma center and the adjacent children’s hospital in the aftermath of the hurricane.

**Methods:**

A retrospective chart review was performed on patients seeking emergent care following the hurricane. Demographic data was abstracted from the medical records along with injury pattern including age, gender, mechanism of injury, total body surface area (TBSA), surgical interventions, and length of stay. In addition, brief surveys of fire chiefs from the two most impacted regions were performed to assess prehospital challenges.

**Results:**

41 patients (76% male) presented to our ER with a median age of 44 (7 patients < 12 years of age). 85% of injuries occurred at home while 15% occurred at work. Of the 78% requiring admission, 66% underwent excision and autograft with a mean TBSA of 17% (range 1-80%). Power outages resulted in increased gas generator usage across the region. Most of the burn injuries following the storm were due to generator and cooking accidents (56%). Each fire chief reported up to 91 calls/day due to suspected carbon monoxide poisoning for the two weeks following the storm. A single event resulted in 8 inhalation injuries treated in our ER with one burn ICU admission. The mean hospital length of stay was 1.11 days/%TBSA for those undergoing surgery.

**Conclusions:**

Hurricanes are more common today with many coastal cities as risk for similar natural disasters. Despite our generator safety media outreach efforts prior to the storm, this remains an opportunity for improved injury prevention. Many patients suffered delays in discharge as their homes/nursing facilities suffered structural damages and were without power and water. Disaster planning should account for limited disposition options during severe storms. Our study is the first to describe burn-related injuries from a category 4 storm and our communities’ response.

